# Development of a loop-mediated isothermal amplification assay combined with a nanoparticle-based lateral flow biosensor for rapid detection of plasmid-mediated colistin resistance gene *mcr-1*

**DOI:** 10.1371/journal.pone.0249582

**Published:** 2021-04-15

**Authors:** Lin Gong, Fei Tang, Ernan Liu, Xiaoli Liu, Huiqiong Xu, Yimei Wang, Yadong Song, Jiansheng Liang

**Affiliations:** 1 Department of Disinfection and Pest Control, Wuhan Center for Disease Control and Prevention, Wuhan, People’s Republic of China; 2 MOE Key Laboratory of Environment and Health, Institute of Environmental Medicine, School of Public Health, Tongji Medical College, Huazhong University of Science and Technology, Wuhan, People’s Republic of China; Nitte University, INDIA

## Abstract

A loop-mediated isothermal amplification assay combined with a nanoparticle-based lateral flow biosensor (LAMP-LFB) was established for the rapid and accurate detection of the mobilized colistin resistance gene (*mcr-1*), which causes the loss of colistin antibacterial efficacy in clinical treatments. The amplification stage of the assay was completed in 60 min at 63°C, and the reaction products could be visually detected by employing the LFB, which provided a fast (within 2 min) and objective method to evaluate the amplification results. The LAMP assay amplified the target sequences of *mcr-1* with high specificity. In pure strains, the detection limit of the LAMP-LFB assay was 360 fg plasmid DNA/reaction, and in spiked feces samples the value was approximately 6.3×10^3^ CFU/mL (~6.3 CFU/reaction), which was tenfold more sensitive than the PCR assay. The results show that the developed LAMP-LFB assay will be a worthy tool for the simple, rapid, specific, and sensitive detection of *mcr-1* gene in clinical settings and resource-limited areas.

## Introduction

As one of the last-resort drugs, colistin is an antibiotic that can treat the serious infections caused by carbapenem-resistant *Enterobacteriaceae* (CRE) [1]. Nevertheless, the global outbreak of CRE has brought about a surged use of colistin, and this phenomenon will inevitably increase the risk of developing colistin resistant bacteria.

In 2015, the understanding of the colistin resistance mechanism involving in chromosomal mutations changed when the plasmid-mediated resistance gene *mcr-1* was discovered [2]. The *mcr-1* was first discovered in China [2], and afterwards which has been detected in various countries including United States of America, Brazil, and Europe [3, 4]. This gene has been identified in different bacterial species, such as *Klebsiella aerogenes* [5], *Citrobacter freundii* [6], *Escherichia coli* [7], *Enterobacter aerogenes* [8], *Klebsiella pneumoniae* [2], *Escherichia fergusonii* [5], *Kluyvera ascorbata* [9]; and *Salmonella enterica* [10]. Not only are *mcr-1*-positive bacteria present in clinical settings (including veterinary hospitals), livestock and vegetable markets, but they can also be isolated from waterborne sources (sea and river water) [11]. The high transferability of the *mcr-1* gene is due to the existence of *mcr-1*-bearing plasmid reservoirs such as IncFI [12], IncFII [13], IncHI1 [14], IncHI2 [15], IncP [16], IncX4 [3] and IncX3-X4 mosaic version [17]. Additionally, the coexistence with other antibiotic resistance genes (KPC, NDM and ESBL) [18–20] broadens the bacteria drug resistance spectrum. The wide dissemination of *mcr-1* could lead to fewer antibiotics being able to treat infections caused by multidrug-resistant isolates. Therefore, the rapid and accurate identification of *mcr-1* could normalize the use of antibacterial agents and reduce the need for empirical therapy.

Recently, conventional and real-time polymerase chain reaction (PCR) assays were employed in the detection of the *mcr-1* gene [21]. Although these common molecular methods possess some merits, such as rapidity and high sensitivity, the requirements of expensive devices and specialized personnel make them unsuitable for primary-care hospitals and “on-site” surveillance [22]. Loop-mediated isothermal amplification (LAMP) is a novel nucleic acid-based technique, known for its reliability, efficiency and rapidity, which has been employed in the detection of genetic material from parasites [23], viruses [24] and bacteria [25]. This approach amplifies DNA (without the denaturing step) at a constant temperature (60–70°C) using Bst DNA polymerase, which triggers the autocycling trait of strand displacement [26]. Due to the isothermal feature, a low-cost water bath can be used to incubate the reaction, instead of an expensive thermocycler [26]. The LAMP-amplified products can be routinely visualized by various means, such as turbidity measurement, electrophoresis, and colorimetric indicator [27]. However, these approaches present some disadvantages. For example, turbidity measurement and electrophoresis require sophisticated equipment, while the colorimetric indicator is unable to detect very low product concentrations. Alternatively, a gold nanoparticles-based lateral flow biosensor (LFB) [28] could be used to detect the amplified target products. Herein, we proposed a LAMP-LFB method to rapidly identify the *mcr-1* gene and evaluated its analytical performance in fecal samples. To the best of our knowledge, it is the first reported LAMP assay coupled with an LFB designed for the screening of *mcr-1*.

## Materials and methods

### Bacterial strains

A total of 21 *mcr-1*-positive and 58 *mcr-1-*negative bacterial strains were used in this study (Table 1). The bacteria carrying the *mcr-1* gene consisted of *Escherichia coli*, *Escherichia fergusonii*, and *Salmonella enteritidis*, for which 13, 7 and 1 isolates were acquired, respectively. Five genotype categories of carbapenemase producers (IMP-4, VIM-1, VIM-2, KPC-2, NDM-5) were incorporated in the non*-mcr-1* isolates: 8 *Escherichia coli*, 28 *Klebsiella pneumoniae*, 2 *Enterobacter cloacae*, and 2 *Pseudomonas aeruginosa*. Similarly, the control group also included non-*mcr-1*/carbapenemase isolates (2 *Escherichia coli*, 4 *Pseudomonas aeruginosa*, 3 *Acinetobacter baumannii*, 2 *Serratia marcescens*, 3 *Staphylococcus aureus*, 3 *Enterococcus faecalis*, and 1 *Enterococcus faecium*). Conventional PCR and subsequent sequencing were used to identify all resistance genes. Genomic, plasmid, and spiked fecal samples DNA were extracted using bacterial genome, plasmid, and stool DNA extraction kits (Tiangen Biotech Co., Ltd., Beijing, China), respectively, according to the manufacturer’s instructions. DNA concentrations were determined using the NanoDrop 2000 (Thermo Fisher Scientific Inc., Waltham, MA, United States). Plasmid DNA extracted from *mcr-1*-producing *E*. *coli* (WHCDC128) was used as a template in the optimization of the LAMP conditions and determination of the detection limits. The LAMP assay was implemented using isothermal amplification kits, which included Bst DNA polymerase and reaction buffer (BeiJing-HaiTaiZhengYuan Technology Co., Ltd., Beijing, China). Centrifuge tubes loaded with reaction mixtures were set in a simple heating thermostat. Afterwards, the LAMP products could be detected via the disposable LFB (BeiJing-HaiTaiZhengYuan Technology Co., Ltd., Beijing, China), the colorimetric indicator Malachite Green (BeiJing-HaiTaiZhengYuan Technology Co., Ltd., Beijing, China), or electrophoresis, for which an UV transilluminator (Analytik Jena AG, Jena, Germany) was used to analyze the gel.

**Table 1:** Bacterial strains.

Genotype	Bacteria species	Strain source^†^	No. of isolates
*mcr-1*	*Escherichia coli*	WHCDC (WHCDC128)	1
	*Escherichia coli*	WHCDC	4
	*Escherichia coli*	ICDC	8
	*Escherichia fergusonii*	ICDC	4
	*Escherichia fergusonii*	WHCDC	3
	*Salmonella enteritidis*	ICDC	1
IMP-4	*Klebsiella pneumoniae*	WHCDC	7
	*Escherichia coli*	WHCDC	5
VIM-1	*Enterobacter cloacae*	WHCDC	2
VIM-2	*Klebsiella pneumoniae*	WHCDC	4
KPC-2	*Klebsiella pneumoniae*	WHCDC	11
	*Pseudomonas aeruginosa*	WHCDC	2
	*Escherichia coli*	WHCDC	3
NDM-5	*Klebsiella pneumoniae*	WHCDC	6
Non*	*Acinetobacter baumannii*	WHCDC	3
	*Pseudomonas aeruginosa*	WHCDC	4
	*Serratia marcescens*	WHCDC	2
	*Escherichia coli*	WHCDC	2
	*Enterococcus faecalis*	WHCDC	3
	*Enterococcus faecium*	WHCDC	1
	*Staphylococcus aureus*	WHCDC	3

*Non, the isolates did not carry the aforementioned genes.

^†^WHCDC, Wuhan Centers for Disease Control and Prevention; ICDC, National Institute for Communicable Disease Control and Prevention, Chinese Center for Disease Control and Prevention.

### Primer design for the LAMP assay

We designed LAMP primers targeting the *mcr-1* gene sequence (GenBank accession number: MK405590.1) using Primer Explore V5 (version 4) and Primer Premier (version 6.0). A cluster of six primers, including one pair of outer primers (F3 and B3), two loop primers (LF and LB), and two inner primers (FIP and BIP), were designed to target several distinct regions of *mcr-1* gene. Moreover, the primers FIP^#^ and LF^#^ originated from the FIP and LF primers, which were labeled with biotin and FITC at the 5’ end, respectively. The primers detailed information is presented in Table 2. All oligomers were purified using HPLC.

**Table 2:** Primers used in the LAMP assay to identify the *mcr-1* gene.

Primers	Sequences and modifications (5’-3’)	Length^†^
LF^#^*	FITC-GCTTACGCATATCAGGCTT	19 nt
LB	ATCGATGGCGTGACCAA	17 nt
FIP^#^*	Biotin-ACGACGAACACCACTAGGCGTAAAGACGCGGTACAAGCAAC	38 mer
BIP	GAGCGCGATACTTTCCCACAGCGCCGCACGATGTGACATT	40 mer
F3	AGTGCGCCAAAAGATACCAT	20 nt
B3	TGAACATACACGGCACAGAA	20 nt

*LF^#^, 5’ end of LF was labeled with FITC. FIP^#^, 5’ end of FIP was labeled with biotin.

^†^nt–nucleotide; mer–monomeric.

### Preparation of gold nanoparticle-based dipstick biosensor

The development of the LFB (4×6 cm) was based on the previous study of Wang and co-workers [29]. A set of four components–absorbent pad, NC membrane, conjugate pad, and sample pad–were orderly assembled on a support card and held together by a plastic casing. The monitoring reagents biotin-bovine serum albumin (2.5 mg/mL) and anti-FITC antibody (0.15 mg/mL) were immobilized on the detection regions of the dry-reagent strips, and formed the CL and TL, respectively. A 5-mm gap was set between the two lines. Cutting machine was used to cut the assembled cards into 4-mm isometric strips. Finally, the assembled biosensors were packed and stored at room temperature, in dry conditions.

### LAMP assay

The LAMP assay was conducted in a 25-μL reaction volume, which contained Bst DNA polymerase (1 μL), colorimetric indicator (1 μL), reaction buffer (12.5 μL), inner primers (1.6 μM), loop primers (0.8 μM), outer primers (0.4 μM), and DNA template (1 μL). The negative controls were preformed using IMP-4-positive *K*. *pneumoniae* (WHCDC261) and KPC-2-producing *E*. *coli* (WHCDC332), while the blank control was done using nuclease-free water. The LAMP mixtures were incubated in isothermal conditions (60–67°C) for 60 min.

Three methods were used to detect the LAMP reaction products: electrophoresis (2% agarose gel), colorimetric indicator, and LFB. In the electrophoretic analysis, the reaction products (4 μL) were drifted at 100 V for 70 min. When using the colorimetric indicator, the color of the reaction mixture containing amplified products remained the same, while in the negative and blank controls the solution turned transparent [30]. When employing LFB, two red lines (CL and TL) could be visualized for the positive reactions, while only the CL was observed in the negative and blank controls [30].

### Specificity and sensitivity of the *mcr-1*-LAMP-LFB method

DNA templates of 21 *mcr-1*-positive and 58 non-*mcr-1* bacterial isolates were used to determine the specificity of the *mcr-1*-LAMP-LFB assay. Colorimetric indicator and electrophoresis were used to confirm the LAMP-LFB assay results, and each assay was performed twice. The sensitivity was determined using tenfold serial dilutions (3.6 ng/μL to 36 fg/μL) of plasmid DNA from the reference strain *E*. *coli* WHCDC128. Measurements were performed three times.

### Evaluation of *mcr-1*-LAMP-LFB assay in spiked feces samples

The performance of the *mcr-1*-LAMP-LFB assay in real matrices was assessed using spiked human fecal samples, which were donated by a consenting adult. The samples were previously screened for the resistance gene, using the PCR assay, and were classified as *mcr-1*-negative. The spike test was carried out as described by Gong and co-workers [30]. Plate counting was performed after the *mcr-1*-producing reference strain *E*. *coli* WHCDC128 was consecutively diluted tenfold (6.3×10^6^–6.3×10^1^ CFU/mL). Further, Afterwards, a fecal sample was inoculated with 100 μL of a diluted culture, this was done for all dilutions. The spiked samples were extracted by a stool DNA extraction kits according to the manufacturer’s specification. The extracted DNA was eluted with 100 μL of buffer solution, and 1 μL of the eluted solution was used in the *mcr-1*-LAMP-LFB assay. The negative control was performed using a non-spiked fecal sample. Finally, three measurements were used for the determination of LAMP products.

### PCR assay

The conventional PCR assay was performed as previously described by Gong and co-workers [30], using 5’-CGGTCAGTCCGTTTGTTC-3’ and 5’-CGGTCAGTCCGTTTGTTC-3’ as the forward and reverse primers, respectively, which were specific for the *mcr-1* gene. The detection threshold of the PCR assay was determined using pure cultures and fecal samples.

### Ethics statement

We utilized the fecal samples from a healthy volunteer in Wuhan Center for Disease Control and Prevention. The volunteer participated in this study when he had signed informed consent. The study protocol was reviewed and approved by the Institutional Review Board of Wuhan Center for Disease Control and Prevention (WHCDCIRB- SQ-2019012).

## Results and discussion

Causing enormous mortality, CRE have become a serious threat in clinical settings. Colistin is recognized as the last line of defense in the treatment of infections caused by those superbugs. However, the antibiotic began to lose its bactericidal effect since the *mcr-1* gene emerged, transmitted, and outbroke in various gram-negative bacteria. Moreover, the *mcr-1* gene carried by transposons and plasmids could bring about no cure when combining with other antibiotic resistance genes, such as KPC and CTX-M, in a single isolate [31]. Thus, the identification of *mcr-1* will contribute to control the gene’s horizontal transmission. Herein, we proposed a loop-mediated isothermal amplification assay coupled with a lateral flow biosensor (LAMP-LFB) to detect *mcr-1* gene.

**Fig 1:**
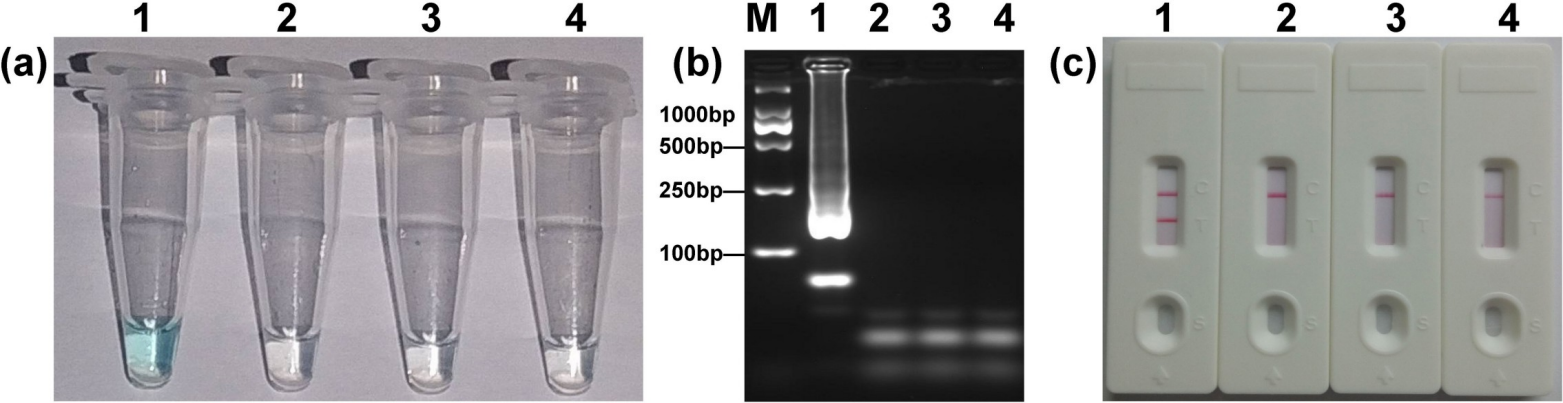
Confirmation of the *mcr-1*-LAMP products. Three detection methods were used to identify the LAMP products: (a) colorimetric indicator; (b) 2% agarose gel electrophoresis; (c) LFB. Four samples were tested: 1. *mcr-1*-producing *E*. *coli* (WHCDC128); 2. IMP-4-positive *K*. *pneumoniae* (WHCDC261); 3. KPC-2-producing *E*. *coli* (WHCDC332); 4. distilled water. The positive results are shown in tube 1, lane 1, and biosensor 1.

A set of *mcr-1* specific primers (Table 2) were designed to target the gene’s conserved region and then screened for the LAMP assay, which was performed under isothermal conditions (62 ^o^C) for 60 min. Amplification products were obtained from *mcr-1*-positive *E*. *coli* (WHCDC128), but not from IMP-4-positive *K*. *pneumoniae* (WHCDC261), KPC-2-producing *E*. *coli* (WHCDC332) and the blank control (Fig 1). Thus, these results confirmed that the three pairs of primers were suitable for the *mcr-1*-LAMP-LFB assay.

In the *mcr-1*-LAMP-LFB system, FIP^#^ and LF^#^ primers (Table 2) were respectively labeled with biotin and fluorescein isothiocyanate (FITC) at the 5’ end. After the LAMP reaction stage, the double-labeled amplicons would be recognized by the anti-fluorescein antibody immobilized in the test line (TL) of the LFB [28]. Further, After adding a small drop (2 μL) of amplification products solution to the biosensor’s test pad, followed by several drops of buffer solution [30], the results could be confirmed visually within 2 min, with the formation of one or two red lines. When compared with electrophoretic monitoring, analysis of LAMP products using LFB is a faster and simpler approach. Additionally, LFB has an accurate judgement when amplicons concentrations are very low, but the color change will be vague in that situation by using colorimetric indicator. Hence, LFB will become a preferable choice for the identification of LAMP products.

**Fig 2:**
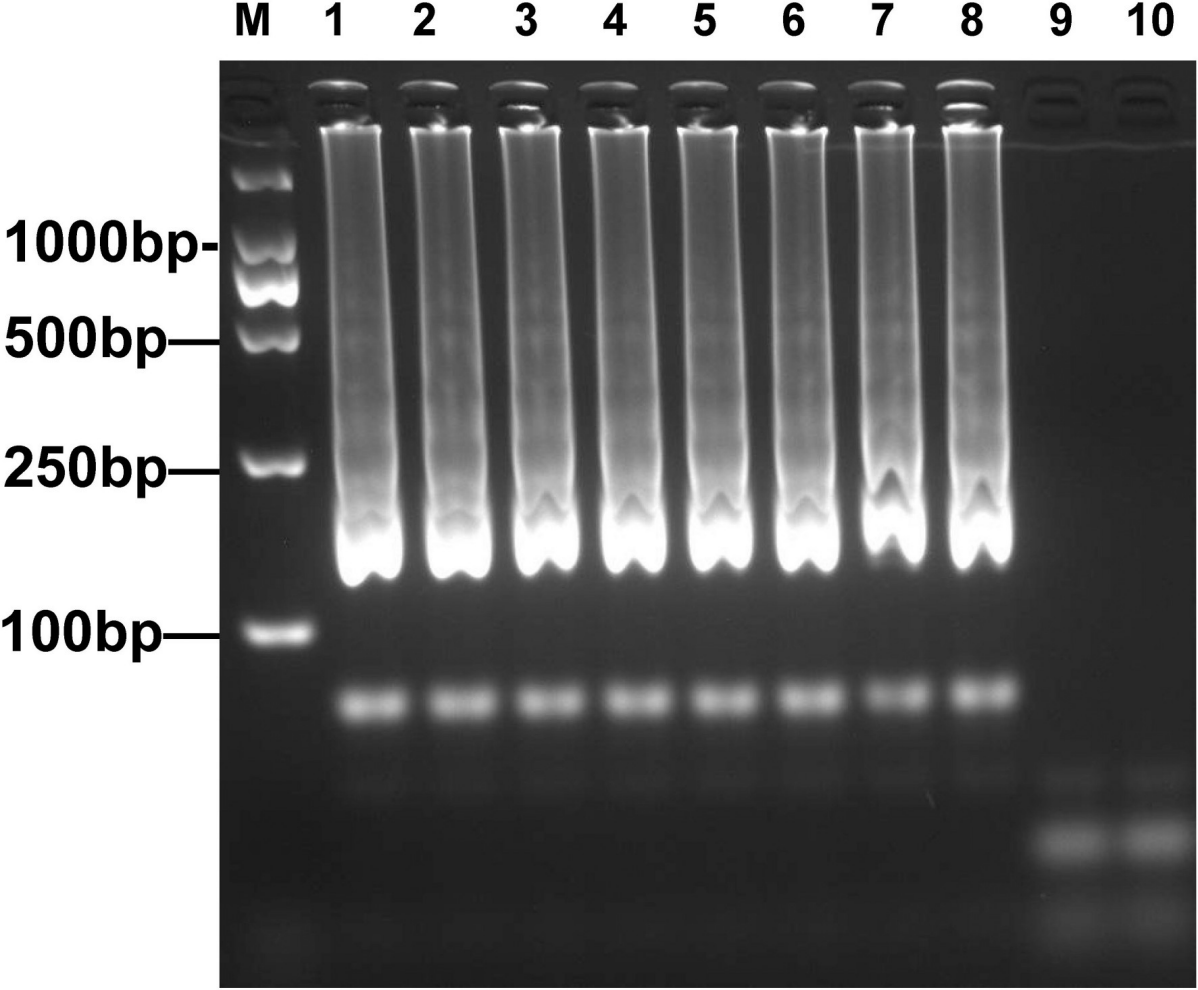
Electrophoretic analysis of *mcr-1* amplicons obtained under different temperature conditions. Lanes 1–8: amplification products generated from *E*. *coli* WHCDC128 plasmid DNA (3.6 pg/μL) with incremented temperatures (60–67°C, 1°C increments). Lane 9: negative control (3.6 pg of *E*. *coli* WHCDC332 genomic DNA). Lane 10: blank control (nuclease-free water).

The optimal reaction temperature will increase the amplification efficiency of the LAMP assay. Therefore, plasmid DNA templates (3.6 pg/μL) from *E*. *coli* WHCDC128 were added into the *mcr-1*-LAMP reaction system in order to optimize the assay’s temperature conditions. Amplicons of *mcr-1* were detected when the reaction was performed at 60–67°C, with 1°C increments for 60 min. According to the electrophoresis results, the best amplification efficiency was obtained in the 62–65°C range (Fig 2), and thus we chose 63°C as the temperature condition to perform further experiments.

**Fig 3:**
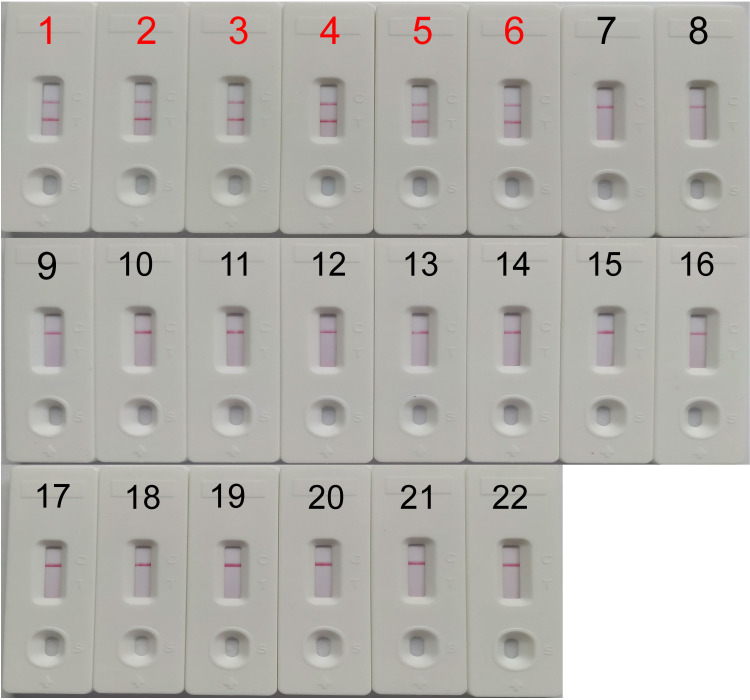
Specificity of the *mcr-1*-LAMP-LFB assay. Biosensors 1–6, *mcr-1*-positive *E*. *coli* WHCDC128, *E*. *coli* from WHCDC, *E*. *coli* from ICDC, *E*. *fergusonii* from ICDC, *E*. *fergusonii* from WHCDC, and *S*. *enteritidis* from ICDC, respectively. Biosensors 7–14, IMP-4-positive *K*. *pneumoniae*, IMP-4-positive *E*. *coli*, VIM-1-positive *E*. *cloacae*, VIM-2-positive *K*. *pneumoniae*, KPC-2-producing *K*. *pneumoniae*, KPC-2-producing *P*. *aeruginosa*, KPC-2-producing *E*. *coli*, NDM-5-positive *K*. *pneumoniae*, respectively. Biosensors 15–21, *A*. *baumannii*, *P*. *aeruginosa*, *S*. *marcescens*, *E*. *coli*, *E*. *faecalis*, *E*. *faecium*, *S*. *aureus*, *respectively* (these isolates did not carry the aforementioned genes). Biosensor 22, blank control (nuclease-free water).

The specificity of the LAMP assay was evaluated by employing LFB (Fig 3). Two red detection bands (TL and control line- CL) were observed for the *mcr-1-*positive strains samples, while only the CL was detected for all the non-*mcr-1* samples, including *mcr-1*/carbapenemase-negative strains, carbapenemase-producing bacteria, and blank control. Therefore, the LAMP assay designed in this study was highly specific for *mcr-1*.

**Fig 4:**
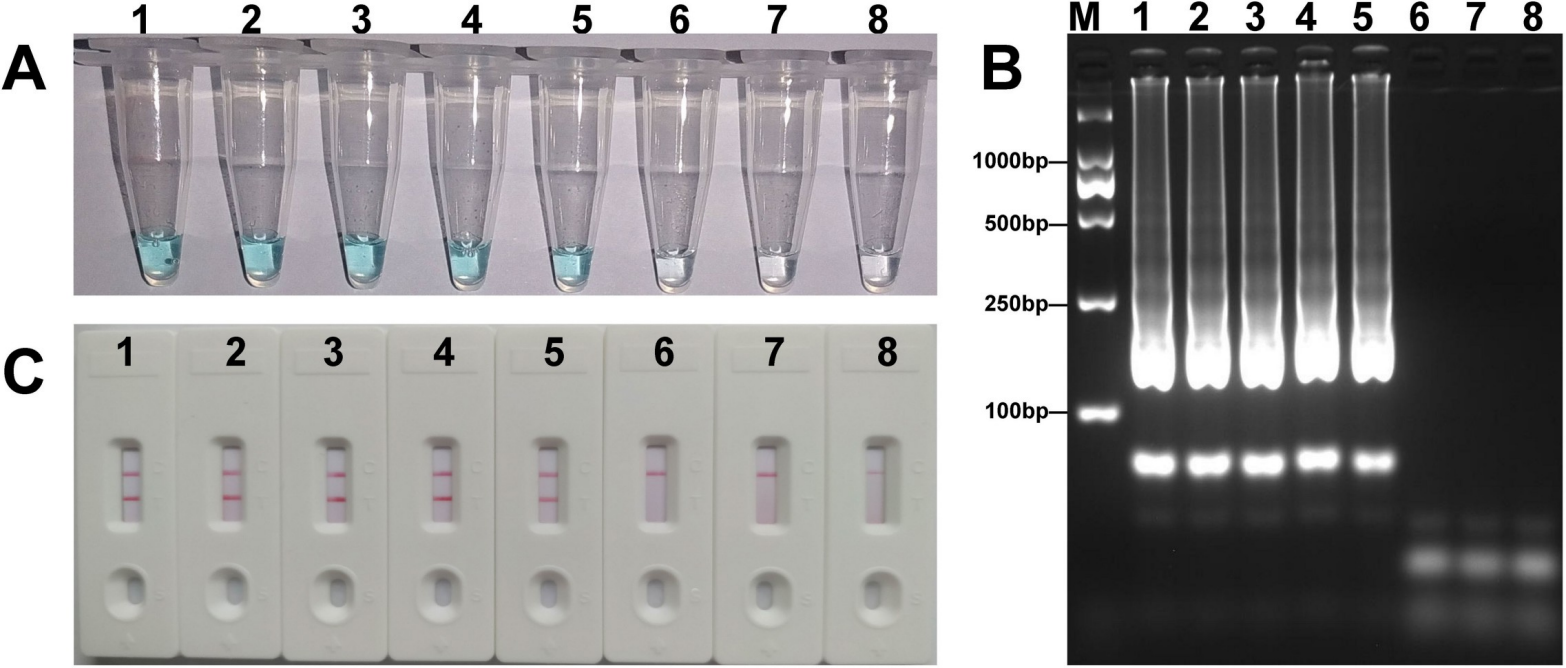
Sensitivity of the *mcr-1*-LAMP-LFB assay. Tubes (A)/lanes (B)/biosensors (C) 1–6 represented the levels of plasmid DNA (*E*. *coli* WHCDC128) 3.6 ng, 360 pg, 36 pg, 3.6 pg, 360 fg and 36 fg per reaction, respectively. Tubes (A)/lanes (B)/biosensors (C) 7 and 8 represented the negative (KPC-2-producing *E*. *coli* WHCDC332) and blank controls (nuclease-free water), respectively.

**Fig 5:**
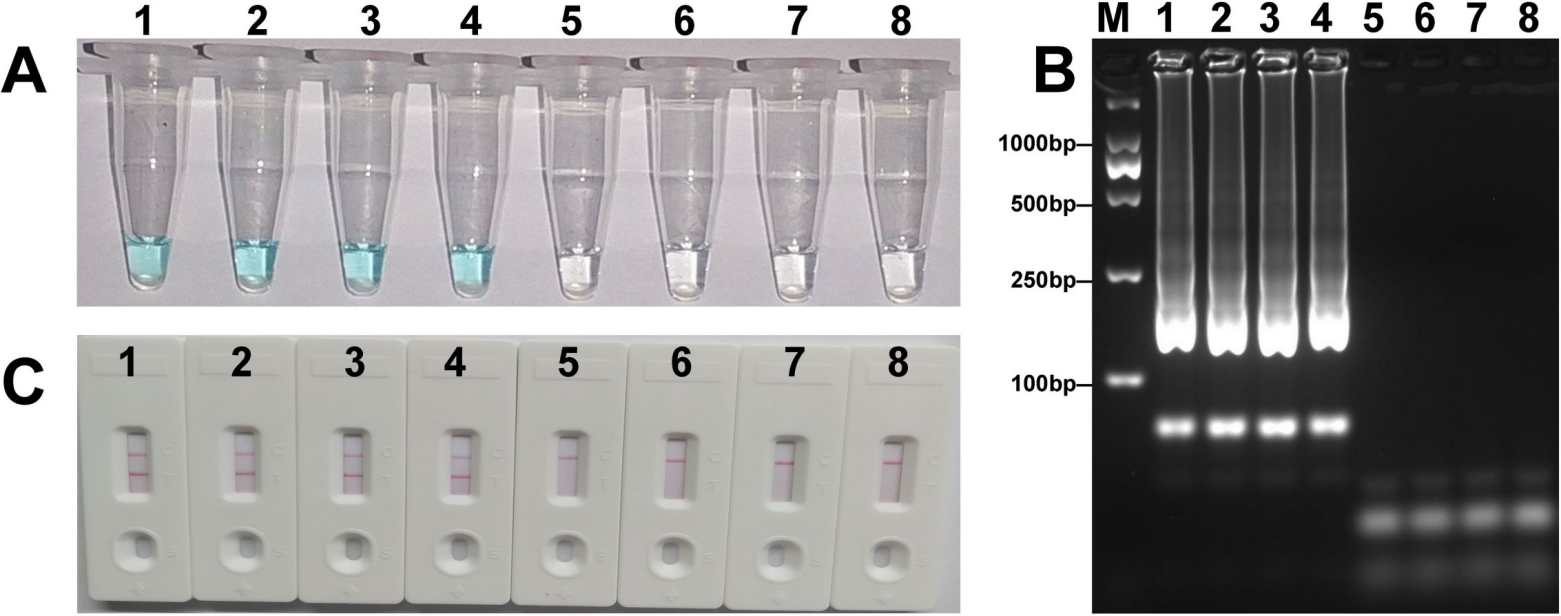
Sensitivity of the *mcr-1-*LAMP-LFB assay in spiked fecal samples. Tubes (A)/lanes (B)/biosensors (C) 1–6 represented the *E*. *coli* WHCDC128 DNA levels of 6.3×10^3^, 6.3×10^2^, 6.3×10^1^, 6.3, 6.3×10^−1^, and 6.3×10^−2^ CFU per reaction, respectively. Tubes (A)/lanes (B)/biosensors (C) 7 and 8 represented the negative (non-spiked fecal sample) and blank controls (nuclease-free water), respectively.

The analytical sensitivity of the *mcr-1*-LAMP-LFB assay was determined using serial dilutions of DNA templates of *E*. *coli* WHCDC128. As shown in Fig 4, LFB analysis indicated that the concentration threshold of the LAMP assay was 360 fg/μL, which was in agreement with the colorimetric indicator and electrophoresis analysis. The practicability of the *mcr-1*-LAMP-LFB was assessed with stool samples inoculated with *mcr-1*-positive isolates. Positive results were observed when *E*. *coli* WHCDC128 concentration was higher than 6.3×10^3^ CFU/mL (~6.3 CFU/reaction) (Fig 5), while lower concentrations, non-spiked sample, and blank control gave a negative result. Similarly, three different approaches obtained identical outcome. Hence, those manifested the established LAMP-LFB approach was suitable for *mcr-1* detection.

The detection limit of the method was 360 fg/L plasmid DNA in pure strains and 6.3×10^3^ CFU/mL in spiked feces samples, which was tenfold more sensitive than the *mcr-1*-PCR assay (Table 3). Moreover, conventional PCR could be inhibited by various interfering substances present in practical specimens, but such inhibitors did not affect the amplification reaction of the LAMP assay [26]. The *mcr-1*-LAMP-LFB assay had the equivalent sensitivity and specificity with the *mcr-1*-MCDA-LFB method described in previously study [30], but the latter was more complex in terms of primers design and screening. Although the detection of *mcr-1* was achieved with high sensitivity using other methods, like MALDI-TOF mass spectrometry and real-time PCR [21, 32], their application in fields of resource shortage would be limited by the need of expensive equipment and rigorous experimental conditions. The *mcr-1*-LAMP-LFB assay overcomes the aforementioned restrictions, requiring only a simple thermostat to supply a constant temperature (63°C), making the assay appropriate for the identification of *mcr-1* on the spot. Importantly, the whole process of the trial, including specimen treatment (20 min), LAMP reaction (60 min) and detection (2 min), could be finished within 85 min. The test time was at least 90 min less than the standard *mcr-1*-PCR method. Therefore, the established method showed distinct merit in the aspect of testing time.

**Table 3:** Conventional PCR and *mcr-1*-LAMP-LFB assay detection limits.

	Detection limit	
	Conventional PCR	*Mcr-1*-LAMP-LFB assay
plasmid DNA	3.6 pg/uL	360 fg/uL
spiked fecal sample	~63 CFU/reaction	~6.3 CFU/reaction

## Conclusion

In conclusion, a LAMP-LFB assay was designed for the identification of the colistin resistance gene *mcr-1*. The method is simple, rapid, sensitive, and specific. The LFB required no additional equipment and could offer a fast and objective way for readout of amplified products. Thus, the *mcr-1*-LAMP-LFB assay will be a worthy tool in clinical settings and resource-poor areas.

## Supporting information

S1 Raw images (PDF)
