# Supplementary figures and images for: Development of a loop-mediated isothermal amplification assay combined with a nanoparticle-based lateral flow biosensor for rapid detection of plasmid-mediated colistin resistance gene mcr-1
Source: PLoS One. 2021 Apr 15;16(4):e0249582. doi: 10.1371/journal.pone.0249582 (PMC8049234; doi:10.1371/journal.pone.0249582)

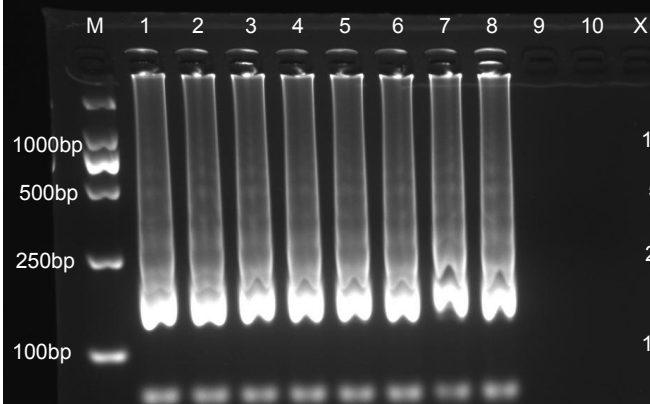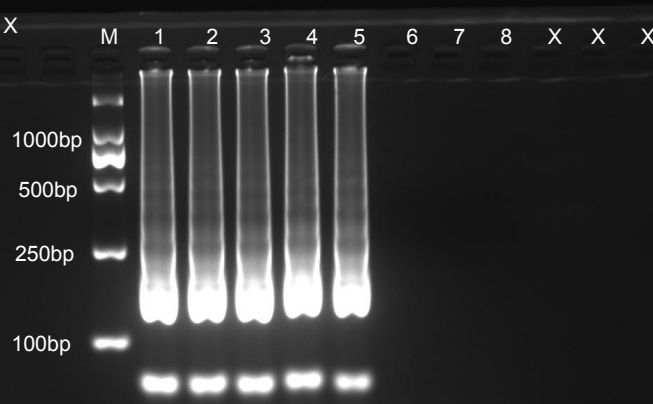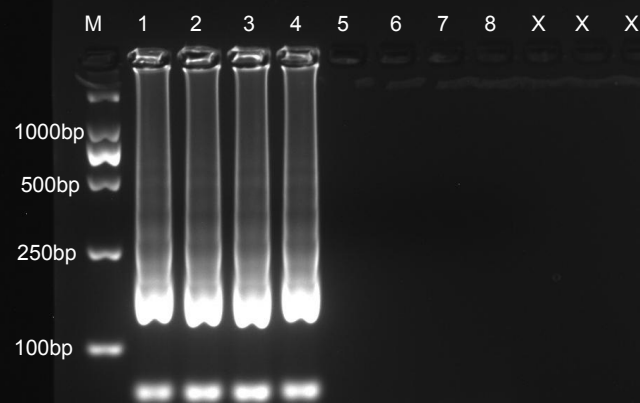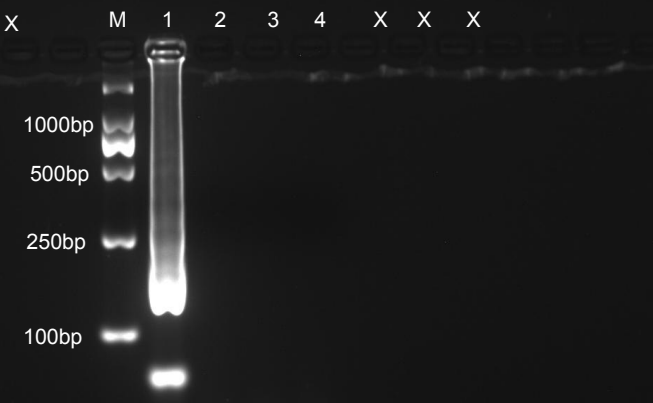

Supplement: S1 Raw images — (PDF) [file pone.0249582.s001.pdf]
